# Occurrence of *Haemoproteus* spp. (Haemosporida: Haemoproteidae) in New Host Records of Passerine Birds from the East of Iran

**Published:** 2018

**Authors:** Leila NOURANI, Mansour ALIABADIAN, Navid DINPARAST DJADID, Omid MIRSHAMSI

**Affiliations:** 1. Dept. of Biology, Faculty of Science, Ferdowsi University of Mashhad, Mashhad, Iran; 2. Malaria and Vector Research Group (MVRG), Biotechnology Research Center (BRC), Pasteur Institute of Iran Tehran, Iran; 3. Research Department of Zoological Innovations, Institute of Applied Zoology, Faculty of Science, Ferdowsi University of Mashhad, Mashhad, Iran

**Keywords:** Haemoproteidae, *Plasmodium*, Blood parasites, Passeriformes

## Abstract

**Background:**

Avian haemosporidians are able to parasitize numerous bird species all over the world. The extensive range of blood parasites infection rate is between 50% and 100% or less percentage. Haemoparasites with major effects on physiology, ecology, health, population dynamics, sexual selection and production success of avian hosts may promote species extinction.

**Methods:**

To evaluate haemosporidians infection rate in Iranian birds, 136 individuals were examined by microscopic observation of stained blood smears under light microscope. These samples belonged to 10 different families of Songbirds from the east of Iran from April to August 2014–2016.

**Results:**

Fifty-one passerine birds were detected as harboring *Haemoproteus* spp. Furthermore, we recorded *Haemoproteus* spp. infection of *Granativora bruniceps*, *Oenanthe pleschanka* for the first time in the world and eight more species for Iran.

**Conclusion:**

Age and sampling localities do not influence the infection rate of *Haemoproteus* spp. from the eastern provinces of Iran. The relative high infection of avian haematozoa revealed this region might provide suitable sites for future studies on these parasites and the relationship with their hosts and vectors.

## Introduction

Various taxa of avian species belonged to passerine birds, the raptors, poultries and migratory birds can act as the possible reservoir of various infectious illnesses that may have an effect on the other animals such as mammals, reptiles and/or human. These probable hosts are able to transfer numerous pathogenic factors including *Plasmodium*, *Leucocytozoon*, feather mite and lice, pox-virus, avian-origin influenza A(H7N9), histoplasmosis, tuberculosis, chlamydophilosis, salmonellosis and psittacosis agents, Arizona infections, Lyme disease and Crimean-congo hemorrhagic fever virus (1–5).

Avian haemosporidians are able to parasitize numerous bird species all over the world (6). The most regular genera of intracellular parasites that can infect bird hosts including *Haemoproteus*, *Plasmodium* and *Leucocytozoon*. Many investigations have declared the extensive range of blood parasites infection rate between 50% and 100% for avian hosts (7, 8) with less percentage of prevalence in some cases (9–11). Avian haemosporidians were in-offensive (12) but others mentioned vast mortality rates (6, 13). Blood parasites with major effects on physiology, ecology, health, population dynamics, sexual selection and production success of avian hosts may promote species extinction (14, 15). The pathogenicity and pathologic changes were caused by different species of haemosporidians (e.g. myositis influence and decrease of growth, anemia, anorexia, ataxia, and death).

*Haemoproteus* as the major genus of haemosporidians with over 140 morphologically distinct species are transmitted by biting midges (Ceratopogonidae) and hippoboscid flies (Hippoboscidae) (6). Several species of *Plasmodium* including *P. relictum* and *P. elongatum* may lead to avian malaria in bird populations. Due to the global distribution of avian heamosporidians, birds may encounter an extensive range of haemosporidian parasites (16). Many investigations have been performed to study the association between prevalence of haemoparasite infections and contributing factors in birds (17–19). In spite of having rich species diversity of Passeriformes in Iran, the infection rate and distribution of avian haemosporidians have been poorly studied (20–22). Blood parasites were considered in free-living pigeons of Columbiforems (11, 23–25), chicken (26), poultry (27), waterfowls (28). Passeriformes are the most recognized clades of birds with more than half of all species distributed worldwide. A recent avian checklist recorded more than 235 passerine species from Iran (29). Concerning the very limited number of studies in this region and the extensive distribution range of these parasites, knowing the prevalence pattern of hemoparasites in various bird hosts seems necessary.

This preliminary study attempted to determine the occurrence of avian haemosporidians in passerine birds from the east of Iran; and, to study the relation between age and sampling locality on the occurrence of *Haemoproteus* spp. Furthermore, new host records of *Haemoproteus* spp. were reported for the first time of this region for Iran and world.

## Materials and Methods

The blood samples were collected from 21 localities in Razavi Khorasan (33° 52′–37° 42′ N and 56° 19′–61° 16′ E), North Khorasan (36° 42′–38° 14′ N and 56° 3′–58° 3′ E) and Semnan (34° 13′– 37° 20′ N and 51° 51′– 57° 3′ E) provinces from Apr to Aug 2014–2016. Birds were caught with mist nets. About 50 μl of whole blood was obtained via brachial vein puncture of the birds with insulin needles. For blood parasites detection, a drop of blood was smeared on three individual microscope slides, then immediately, air-dried, fixed in absolute methanol and finally stained with Giemsa stain, pH 7.2. In order to specify the infection rate of haematozoa, each slide was screened in 150–180 fields under low magnification (×400), and at least 100 fields at high magnification (×1000) with immersion oil under Olympus BH2 light microscope (Olympus Co, Japan). For parasites identification, haemosporidians gametocytes were observed inside red blood cells according to the proposed procedures (6). All birds were released after collecting blood samples. Animal care and experimental procedures were achieved in accordance with the guidelines and protocols permitted by the ethics committee for the care and use of animals for scientific purposes of the Biology Department, Ferdowsi University of Mashhad, Iran. The significant differences in occurrence between hostage (mature and immature) and sampling localities were analyzed using Pearson Chi-square test (*P*<0.05) on SPSS ver. 16 (Chicago, IL, USA).

## Results

Overall, 136 individuals (from 20 species) were examined for blood parasite presence. All samples belonged to the order Passer-iformes comprising 10 families: Turdidae, Paridae, Passeridae, Emberizidae, Fringillidae, Acrocephalidae, Phylloscopidae, Motacillidae, Muscicapidae, and Hirundinidae. The most common captured family was Passeridae (51.5%). Razavi Khorasan was nominated as the most frequent place of capture (64.7%), with North Khorasan (24.3%) and Semnan (11%) following in the subsequent order. The proportion of immature to mature hosts was 15.4% and 84.6% in our specimens, respectively.

### Infection rate of Haemoproteus spp. in passerine hosts

**Fig.1:**
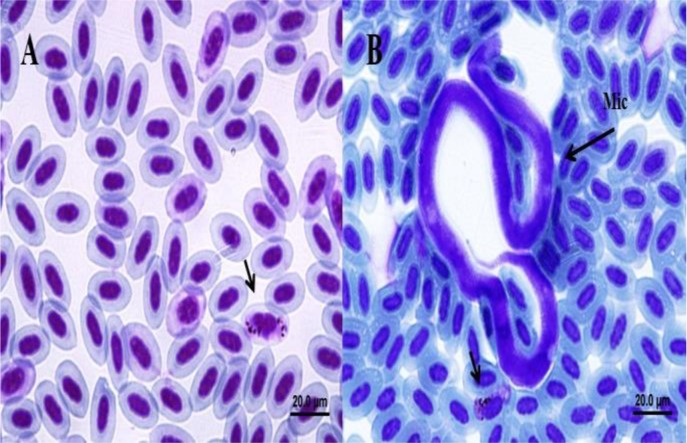
Macrogametocyte of *Haemoproteus* spp. in *Oenanthe pleschanka* are shown in part A and Macrogametocyte and Microfilariae of *Granativora bruniceps* from east of Iran presented in part B (Photo by L. N.)

Fifty-one hosts (37.5% overall infection) were detected as harboring *Haemoproteus* spp. representing 11 species of *Passer domesticus, P. montanus, Granativora bruniceps, Fringilla coelebs, Carduelis carduelis, Acrocephalus dumetorum, A. stentoreus, Iduna pallida, Motacilla alba, Oenanthe pleschanka,* and *Hirundo rustica* (Table 1). The families of Passeridae, Emberizidae, Fringillidae, Acrocephalidae, Phylloscopidae, Motacillidae, Muscicapidae and Hirundinidae harbored *Haemoproteus* spp. in which Passeridae was positioned as the most infected family (19.85%). The most infected species to *Haemoproteus* infection were *Passer domesticus* and *Acrocephalus stentoreus*. The infection rate of age categories for *Haemoproteus* spp. showed a higher infection rate for mature (39.13%) in comparison with immature hosts (28.57%) (*P*<0.05), however, there were not any significant differences between age categories for *Haemoproteus* infection (*P*= 0.358). The infection rate of *Haemoproteus* for North Khorasan, Razavi Khorasan and Semnan was 48.5%, 35.2% and 26.7%, respectively (*P*<0.05) without any significant differences between sampling areas (*P*=0.267). Furthermore, we recorded *Haemoproteus* spp. infection of *Granativora bruniceps* and *Oenanthe pleschanka* for the first time in both the world and Iran (Fig. 1).

**Table 1:** Occurrence of *Haemoproteus* spp. in passerine birds from east of Iran

***Host family and species***	***n***	***H***	***H (%)***	***Hae***
**Turdidae**				
*Turdus merula*	1 (R)	-	0	2
**Paridae**				
*Parus major*	4 (R)	-	0	9
**Passeridae**				
*Passer domesticus* ^∞^	10 (K), 4 (S), 22 (R)	8 (K), 2 (S), 16 (R)	50.98	9
*Passer montanus* ^∞^	3 (K), 31 (R)	1 (R)	1.96	3
**Emberizidae**				
Granativora bruniceps **	6 (K)	1 (K)	1.96	0
**Fringillidae**				
*Fringilla coelebs* ^∞^	1 (R)	1 (R)	1.96	9
*Carduelis carduelis* ^∞^	1 (K), 1 (R)	1 (R)	1.96	1
*Carduelis flammea*	1 (R)	-	0	5
*Erythrina erythrinus*	1 (K)	-	0	7
**Acrocephalidae**				
*Acrocephalus dumetorum* ^¥^ ^∞^	6 (R)	5 (R)	9.8	2
*Acrocephalus stentoreus* ^∞^	2 (K), 10 (R)	1 (K), 6 (R)	13.72	1
*Iduna pallida* ^∞^	1 (R), 8 (K)	6 (R)	11.76	5
**Phylloscopidae**				
*Phylloscopus trochilus*	1 (S)	-	0	6
*Seicercus trochiloides*	1 (S)	-	0	6
*Seicercus nitidus*	1 (R)	-	0	3
**Motacillidae**				
*Motacilla alba* ^∞^	1 (K), 2 (S)	1 (S)	1.96	1
**Muscicapidae**				
*Saxicola torquatus*	1 (K)	-	0	0
Oenanthe pleschanka **	7 (S)	1 (S)	1.96	0
**Hirundinidae**				
*Hirundo rupestris*	2 (R)	-	0	0
*Hirundo rustica* ^∞^	7 (R)	1 (R)	1.96	10
**Total**	136	51 (37.5%)		

n= number of examined host, H= *Haemoproteus*, H (%)=*Haemoproteus* prevalence, and Hae= number of recorded lineage of Haemoproteus in MalAvi database, respectively. K= North Khorasan, R= Razavi Khorasan, and S= Semnan provinces

∞ New host records for Iran,

** new record for world and Iran, and

¥ new host record for northwest of Iran (22)

## Discussion

Avian blood parasites are already distinguished by the morphological features in more than 140 species of *Haemoproteus*, 50 different species of *Plasmodium* and over 35 species of *Leucocytozoon* (30). These parasites could affect hosts in the extensive range from modified physiology to death (14, 15). The pathogenicity and pathologic changes caused by haemosporidians have demonstrated the importance of identification of these blood parasites in various hosts. In order to confirm the pathogenicity of *H. meleagridis*, an experimental study by sporozoite-induced infections showed the serious myositis influence and decrease of development in domestic turkey poults (31). In another study, *H. danilewskyi* experimental infection in captive and naturally infected *Cyanocitta cristata* showed the raised numbers of WBCs and reduced PCV rate. Besides, sublethal pathologic modifications were detected in the spleen, lung, and liver (32).

The pathogenicity of numerous species of Leucocytozoidae was declared as the evidence for high rate of death in the domestic chickens and other poultry (6). For instances, the *L. caulleryi* infection may lead to the intensive symptom of anemia, anorexia, ataxia, and even sever problem in taking breath in chickens. The hemorrhages caused by separation of megalomeronts and growth in tissues may lead to boost the mortality rate in birds (33).

*Haemoproteus* as a vector-borne parasite is able to parasitize more than 67% avian hosts globally (6). Our results demonstrated that 11 species were infected with *Haemoproteus* spp. Except for *A. dumetorum,* recorded from the northwest of Iran (22), the remaining 10 identified hosts were new records of *Haemoproteus* infection from the eastern part of Iran. According to the MalAvi database, until Aug 2016, no *Haemoproteus* spp. infection was reported in any of the species that comprised the specimens within this study. Our study recorded *Haemoproteus* spp. infection in two species of *Granativora bruniceps* (North Khorasan), *Oenanthe pleschanka* (Semnan) for first time in the world. Extensive research has documented the prevalence rate of haemosporidian infection in birds across the world.

The infection rate of haemosporidian in Columbiformes, Anseriformes and crow species (Passeriformes) from Japan was 21%, 17%, and 93.8%, respectively (8). Our results were similar to recent studies on European passerine birds infection rates with an average of 26% for haematozoa in 14812 specimens (9). Estimation of hematological values in Shiraz, Iran, demonstrated 14.32% of indigenous chickens were infected with *P. gallinaceum* (26). *Haemoproteus* as the most common genus of blood parasite in birds was discovered in 37.5% of our samples. Moreover, we could not detect any closely related genera of haemosporidians (*Plasmodium* and *Leucocytozoon*) in our specimens from these sampled areas. These results may verify the presence of Hippoboscidae and/or Ceratopogonidae as the major vectors of *Haemoproteus* spp. in birds from these regions. According to last researches on these mosquito’s families in Iran (34–36) and concerning the fact that mosquitos biting and blood feeding may lead to transfer some disease agents to animals and man (37), more studies on the dipteran fauna of prevalent area seems to be essential.

*Appraisal of Haemoproteus* spp*. infection with age and locality*

Prevalence is a complex parasite parameter and seems to be influenced by a great number of factors such as the age, sex, habitat, altitude, the immune system, and so on. In this study, two factors of locality and age categories were analyzed with the infection rate of detected hemoparasites. Although more than 84.6% of our specimens were mature, no significant tendency in *Haemoproteus* infection was observed between the age categories. Furthermore, the infection rate for *Haemoproteus* spp. of each province identified North Khorasan (48.5%) as the prevalent area, however statistical analysis failed to show any significant difference between sampling localities and *Haemoproteus* spp. infection. In a similar study on the infection rate of avian blood parasites of *Lanius meridionalis*, no significant differences were reported between age, sex and season for the infected hosts (38). Conversely, the presence of a relationship between age and malaria infection was specified in *Cygnus olor* (39). An examination on *Neothraupis fasciata* from central Brazil indicated age as a significant factor in the infection of on haemosporidians infection (40). In eastern Russia, the prevalence of *Haemoproteus* (31.4%), *Leucocytozoon* (33.1%), *Plasmodium* (12.4%), and microfilariae (8.9%) were documented, respectively and there was no apparent difference in the prevalence of infection between host sexes or between young and adult birds (41). Due to the opponent idea about the relationship of haemosporidians and age categories and locality, the presence or absence of a significant relationship diversifies for each avian species per locality with different habitat or climate.

## Conclusion

Age and sampling localities do not influence the infection rate of *Haemoproteus* spp. from the eastern provinces of Iran. The relative high infection rate of avian haematozoa in passerine birds revealed this region might provide suitable sites for future studies on these parasites and the relationship with their hosts and vectors. Due to the prominence of transmission of diseases between wildlife and man, having an effect on public health and economic issues for example in poultries, pets, and ornamental birds, and the probable high infections with wild hosts, more investigations may shed light on the birds and parasites association.
